# Development of a high-throughput in vitro screening method for the assessment of cell-damaging activities of snake venoms

**DOI:** 10.1371/journal.pntd.0011564

**Published:** 2023-08-17

**Authors:** Matyas A. Bittenbinder, Liliana Capinha, Daniel Da Costa Pereira, Julien Slagboom, Bas van de Velde, Nicholas R. Casewell, Paul Jennings, Jeroen Kool, Freek J. Vonk

**Affiliations:** 1 Naturalis Biodiversity Center, Leiden, The Netherlands; 2 AIMMS, Division of BioAnalytical Chemistry, Department of Chemistry and Pharmaceutical Sciences, Faculty of Sciences, Vrije Universiteit Amsterdam, Amsterdam, The Netherlands; 3 Centre for Analytical Sciences Amsterdam (CASA), Amsterdam, The Netherlands; 4 Division of Molecular and Computational Toxicology, Department of Chemistry and Pharmaceutical Sciences, Vrije Universiteit Amsterdam, Amsterdam, The Netherlands; 5 Centre for Snakebite Research & Interventions, Liverpool School of Tropical Medicine, Liverpool, United Kingdom; Monash University, AUSTRALIA

## Abstract

Snakebite envenoming is a globally important public health issue that has devastating consequences on human health and well-being, with annual mortality rates between 81,000 and 138,000. Snake venoms may cause different pathological effects by altering normal physiological processes such as nervous transfer and blood coagulation. In addition, snake venoms can cause severe (local) tissue damage that may result in life-long morbidities, with current estimates pointing towards an additional 450,000 individuals that suffer from permanent disabilities such as amputations, contractions and blindness. Despite such high morbidity rates, research to date has been mainly focusing on neurotoxic and haemotoxic effects of snake venoms and considerably less on venom-induced tissue damage. The molecular mechanisms underlaying this pathology include membrane disruption and extracellular matrix degradation. This research describes methods used to study the (molecular) mechanisms underlaying venom-induced cell- and tissue damage. A selection of cellular bioassays and fluorescent microscopy were used to study cell-damaging activities of snake venoms in multi-well plates, using both crude and fractionated venoms. A panel of 10 representative medically relevant snake species was used, which cover a large part of the geographical regions most heavily affected by snakebite. The study comprises both morphological data as well as quantitative data on cell metabolism and viability, which were measured over time. Based on this data, a distinction could be made in the ways by which viper and elapid venoms exert their effects on cells. We further made an effort to characterise the bioactive compounds causing these effects, using a combination of liquid chromatography methods followed by bioassaying and protein identification using proteomics. The outcomes of this study might prove valuable for better understanding venom-induced cell- and tissue-damaging pathologies and could be used in the process of developing and improving snakebite treatments.

## Author summary

Snakebite envenoming is a neglected tropical disease with a global scale that results in over 100,000 fatalities per year and morbidity rates surpassing 450,000 individuals. This study focuses on the mechanisms behind venom-induced tissue damage, more specifically on cytotoxicity and extracellular matrix degradation. We applied a combination of cellular assays and fluorescent microscopy to study the effects of crude and fractionated venoms of a selection of medically relevant snake species. Our findings reveal a notable difference in the way in which viper venoms and elapid venoms exert their effects on cells. The study comprises both morphological data as well as quantitative data on cell metabolism and viability over time. This data could be valuable for better understanding venom-induced cell damage and may prove useful in the process of developing and improving treatments for (local) tissue damage.

## Introduction

Snakebite is one of the most significant global health crises known to date with severe implications for human health and well-being, especially in the tropical and subtropical countries of the developing world. Worldwide, snakebites are accountable for mortality rates ranging from 81.000 to 138.000 individuals each year [1]. As most cases of snakebite affect male agricultural workers and occur in developing countries in sub-Saharan Africa, South and Southeast Asia, and Latin America, the socioeconomic impact of snakebite on families and local economies can be substantial [1–5].

Snake venom is a highly variable mixture of dozens of enzymatic and non-enzymatic proteins which may produce a range of local and systemic effects in bite victims, with some being life-threatening while others being permanently debilitating [1,6]. The clinical effects of snakebite envenoming can be divided into three main pathologies: haemotoxicity, neurotoxicity and tissue damaging effects [1,7,8]. Although most life-threatening pathologies in snake bite victims result from the haemotoxic and neurotoxic effects, the tissue damaging activities are the main cause of life-long disabilities such as blindness and amputations [1–3,9]. Although annual morbidity rates surpass 450,000 individuals, most research has been focusing on the haemotoxic and neurotoxic effects and considerably less on venom-induced tissue damage [1,10]. The cell- and tissue damaging effects of snake venoms may result in a range of pathologies including (local) haemorrhage, muscle and skin necrosis, oedema and organ failure [1,8,11–13].

Tissue damaging toxins can be loosely divided in two groups based on the mechanisms of actions by which these toxins affect the cells. The first group are toxins are ‘true’ cytotoxins, as these directly affect the cells by disrupting cell membrane integrity. These include three-finger toxins (3FTxs), phospholipases A_2_ (PLA_2_) and β-defensin-like toxins [1,7,12–17]. The second group of toxins could be considered to be indirectly cytotoxic and cause cell death as a secondary effect of the degradation of extracellular matrix (ECM) components, not by directly damaging the cells. This group consists of snake venom metalloproteases (SVMPs) and hyaluronidases [11,18–20]. Other toxins that may cause cell- or tissue damaging through alternative mechanisms effects include disintegrins, L-amino acid oxidases (LAAOs) and possibly C-type lectins [1,21–23].

**Fig 1:**
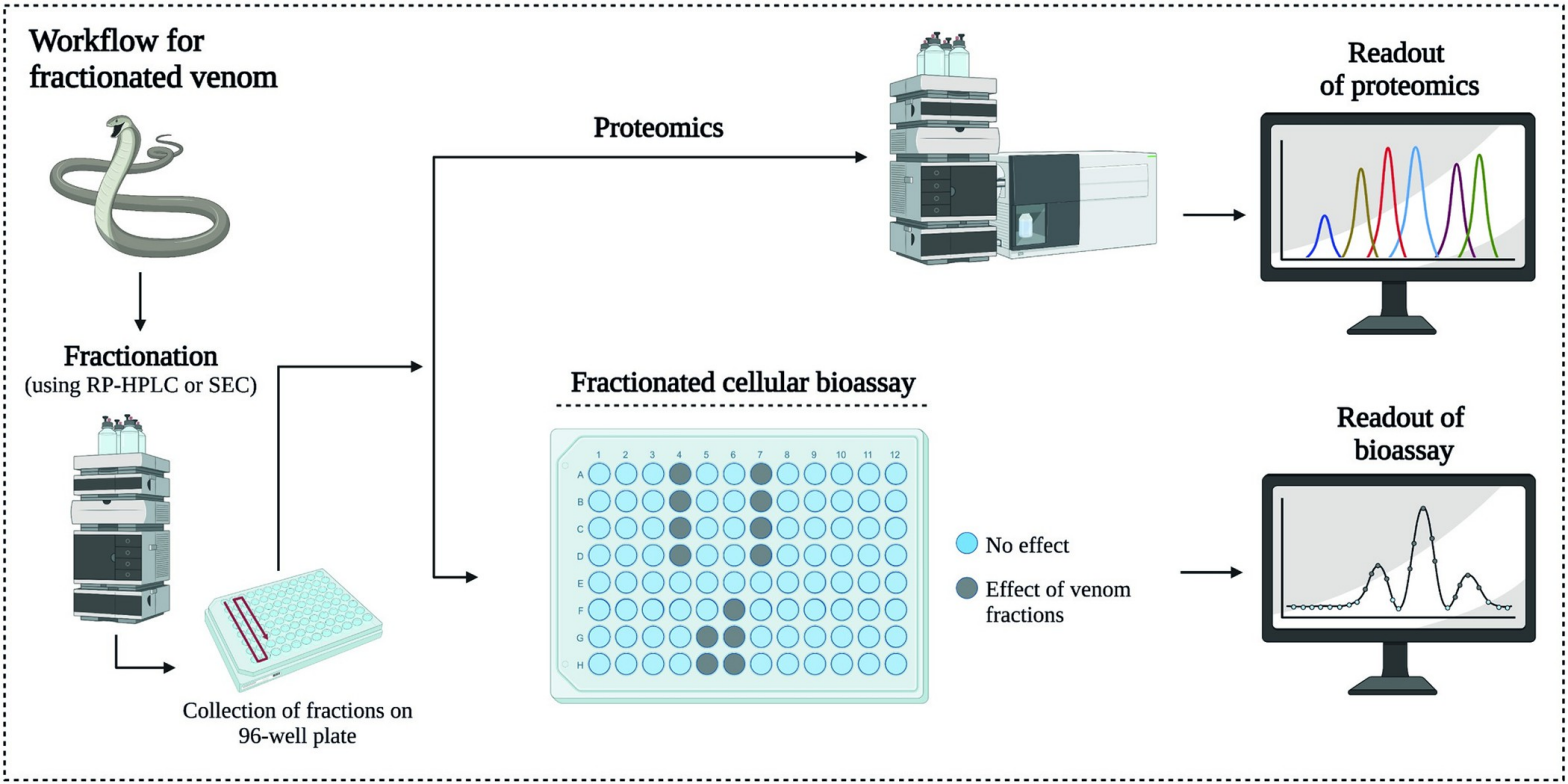
Graphical overview of the bioassay workflow. After injection of the venom, chromatographic separation by HPLC is performed, followed by high-resolution fractionation on 96- or 384-well plates for subsequent cellular bioassaying and protein identification using proteomics as described by Slagboom et al. [27]. Image created using www.biorender.com.

In an attempt to study venom-induced cell damage and the toxins associated with it, we combined a panel of cell-based assays with nanofractionation analytics. First, a panel of cellular bioassays was used to study the effects of ten medically relevant snake venoms *in vitro*, using an immortalised human renal proximal tubular cell line (RPTEC/TERT1). We investigated venom effects over time using an imaging fluorescent microscope, which was used to study both morphological alterations of the cells and quantification of the total live cell count and cell surface area. In addition, we performed assays to determine the metabolic activity and cell viability of the cells post-venom dosing. Based on these assays, a distinction could be made in the way by which the venoms of elapid and viper venoms affect the cells. The venoms of *N*. *mossambica* and *N*. *naja* affected the cell membrane, whereas all vipers (except *D*. *russelii*) caused detachment of the cell monolayer. The assays were then used for integration into nanofractionation analytics. In nanofractionation analytics, chromatographic separation of a venom by HPLC (i.e. reversed-phase chromatography (RP-HPLC) or size-exclusion chromatography (SEC)) is followed by toxin fractionation on 96- or 384 well plates for subsequent (cellular) bioassaying and toxin identification [24–26]. This workflow allowed us to identify toxins with cytotoxic properties in the venom of the Mozambique spitting cobra (*Naja mossambica*) and West African carpet viper (*Echis ocellatus*). Subsequently, we used proteomics for detailed characterisation analysis whereby wells containing bioactive compounds were subjected to tryptic digestion followed by nanoLC-MS/MS for protein identification. Incorporation of the *in vitro* assays into the established analytical workflow allowed for the separation of the toxins followed by bioactivity assessment in parallel with toxin identification (Fig 1).

This approach allowed us to perform high-throughput profiling of cell- and tissue damaging toxins in snake venoms. The outcomes of this study could be particularly useful for the future development of snakebite treatments against venom-induced tissue damage.

## Methods

### Venom preparation

Venoms were sourced from the extensive library of the Faculty of Science, BioAnalytical Chemistry, Vrije Universiteit Amsterdam (VU). This library contains samples obtained and subsequently provided by the Liverpool School of Tropical Medicine (LSTM), National University of Singapore (NUS) and by captive breeders. The snake venoms used in this study came from the following viper (Viperidae) and elapid (Elapidae) species: *Bitis arietans* (puff adder, captive bred), *Bothrops jararaca* (jararaca, captive bred), *Bungarus multicinctus* (many-banded krait, locality unknown), *Calloselasma rhodostoma* (Malayan pitviper, Thailand), *Daboia russelii* (Russell’s viper, locality unknown), *Echis carinatus* (Indian saw-scaled viper, India), *Echis ocellatus* (West African carpet viper, Nigeria), *Naja haje* (Egyptian cobra, captive bred), *Naja mossambica* (Mozambique spitting cobra, captive bred) and *Naja naja* (Indian cobra, captive bred). These species were selected as they represent some of the most medically relevant species across the geographical regions most heavily affected by snakebite (i.e. Latin America, Sub-Saharan Africa and Southeast Asia). Venoms from VU and NUS were lyophilised immediately after milking, then freeze-dried and stored at -80°C. LSTM venoms were extracted, stored overnight at -20°C, then lyophilised and stored at 4°C for long term. Samples were reconstituted in milliQ-water (mQ) to the desired stock solutions, depending on the type of assay. These solutions were then aliquoted and subsequently snap-frozen in liquid nitrogen and stored at -80°C until use. All venoms were sourced in accordance with the Nagoya protocol, where applicable [28].

### Cell culture

The RPTEC/TERT1 cell line was used to study the cell-damaging effects of our panel of medically relevant snake venoms. Cells were routinely cultured on 96-well flat bottom plates (Greiner Bio-One) according to a protocol by Jennings et al., 2009 [29].

Cells were exposed to increasing venom concentrations of ten medically relevant snake species for 3, 6, 12, 24 and 48 hours or 0.1% Triton T-100 as positive control. A five-point serial dilution was generated of decreasing venom concentrations: 100 μg/mL, 33.3 μg/mL, 11.1 μg/mL, 3.7 μg/m and 1.2 μg/mL. In each well the concentration of the diluted venom relative to the medium was 1:9 and the negative control was accordingly incubated with a 1:9 dilution of mQ in medium [30].

### Fluorescent microscopy

Morphological data was collected using a confocal Operetta CLS high content imager (PerkinElmer) with 10X, 20X or 63X objectives and a brightfield channel (white light), a digital phase contrast (DPC) channel and two fluorescence channels. Although the brightfield channel is readily available in most microscopes the limitation with this channel is the fact that the contrast is often poor making visual cell analysis challenging [31]. Therefore, an additional channel was applied using digital phase contrast, which is a contrast-enhancing optical technique that can be used to produce high-contrast images of transparent subjects such as living cells. The combination of these four channels allowed us to closely follow the effects that the venoms had on the cells.

We employed several methods to quantify the cell-damaging activities caused by the venoms. In order to calculate the live cell count we made use of two chromosome stains, Hoechst 33342 (H342)–a cell permeable dye that is often used for staining cell nuclei–and propidium iodide (PI), which is impermeable for live cells. The latter functions as a counterstain due to its inability to cross the intact plasma membrane and is an ideal marker for destabilisation of the cell membrane. Once PI is bound, its fluorescence increases 20–30 fold [32]. H342 (Thermo Fisher Scientific) was used at a concentration of 1:20,000 and PI (Sigma-Aldrich) was used at 1:10,000, both stains were diluted in growth medium and protected from light. Cells were subsequently incubated for 15 min at 37°C with the medium containing both stains before addition of the venoms. The cells were then imaged using two fluorescence channels: Ex355-385 nm Em430-500 (for H342) and Ex530-560 nm Em570-650 nm (for PI). Images were collected using Harmony software 4.8 (PerkinElmer). The software was used to quantify the total cell count (cells stained with H342) and the number of dead cells (stained with PI) (for details, see S8 Fig).

To obtain the live cell count we subtracted the number of dead cells from the total cell count. We then divided the number of live cells by the negative control, yielding the percentage of live cells relative to negative control.

For the venoms capable of disrupting the ECM and thereby detaching the cell monolayer from the bottom of the well, we used the brightfield channel to quantify the surface area of the cell monolayer. For this we again used the Harmony software, this time to calculate the surface area of cell relative to ‘empty’ areas in the wells, in those spots where the monolayer was (partially) subtracted. The software was employed to differentiate cell populations from regions without cells (i.e. ‘empty well area’) using a simple learn-by-example approach (for details, see S9 Fig). The machine-learning technology available in the software was then used to generate an image analysis algorithm that enables quantification of cytotoxicity in terms of ECM-degradation.

### Resazurin reduction assay

Cell metabolism was studied by subjecting the cells to a colorimetric assay that uses Alamar Blue reagent. This assay is used to quantify the number of cells with active metabolism and is based on the reduction of resazurin, a non-fluorescent indicator which is metabolised to its fluorescent metabolite resorufin by viable cells in culture. At the end of the exposure time (i.e. 3, 6, 12, 24 or 48 hours), 5 μL of Alamar Blue (Thermo Fisher Scientific) was added to each well and incubated for 1 hour. The fluorescence was subsequently measured using a CLARIOstar Plus reader (BMG Labtech) at 540 nm for excitation and 590 nm for emission. Cell metabolism was calculated by comparing the fluorescent values of the venom samples with those of the negative control (i.e. average signal without venom, where normal metabolic activity is expected).

### ATP assay

Cell viability was quantified using ATP-levels in cell lysates. As ATP is involved in a variety of enzymatic reactions and ATP-levels decline rapidly when cells undergo necrosis or apoptosis, this assay is widely accepted as a valid marker of viable cells, as higher ATP-concentrations indicates a higher number of living cells. This bioluminescent assay uses ATP from viable cells to generate photons of light. ATP-levels were measured using the ATPlite kit from PerkinElmer (6016731) according to the manufacturer’s instructions. After the cells were incubated with the venom for 24 or 48 hours, cells were subjected to this assay after the luminescence was measured on a CLARIOstar Plus reader (BMG Labtech). Cell viability was subsequently quantified by comparing the ATP-levels in venom-incubated wells with the ATP-levels of the negative control (where cells were 100% viable).

### Liquid chromatography, nanofractionation and mass spectrometry

In order to investigate which venom toxins could potentially cause cell-damaging effects, we used advanced analytical methods to separate the crude venoms in to their individual toxins. For this, we used the method developed by Slagboom et al. [33]. Section 1 in the S1 Methods provides a more elaborate description of this separation method.

### Correlating bioassay data with toxin identification

For subsequent analysis of the fractions, we selected a representative species of each snake family of our panel of snake venoms. Three concentrations of crude venom were used for separation onto the 96-wells plates: 0.5; 1.0; 2.5 mg/mL. These plates were then subjected to the same panel of cell bioassays as described above. Venoms were separated with RP-HPLC followed by fractionation on 96-well plates for subsequent cellular bioassaying and toxin identification. The obtained bioactivity data was converted into bioactivity chromatograms by plotting the readout of each well on the Y-axis against the elution time of each fraction in each well on the X-axis.

In order to analyse the bioactive fractions in *E*. *ocellatus* venom (for which the toxin activity was affected by our ‘traditional’ separation method) we applied an alternative approach using SEC with an eluent based on non-volatile salt buffers with DPBS as the mobile phase. Two concentrations of *E*. *ocellatus* venom (i.e. 2.5 and 5.0 mg/mL) were fractionated onto 96-well plates. With this approach, we were able to collect the venom toxins in their native form. Section 2 in S1 Methods provides a more elaborate description of this method.

### Identification of bioactive compounds by post-column proteomics

Following the identification of wells containing bioactive compounds, venom fractions were subjected to tryptic digestion in order to identify bioactive fractions using proteomics. For this, the proteomics approach developed by Slagboom et al. (2023) was used which was adapted to a 96-well plate [27]. Section 3 of S1 Methods provides an extensive explanation of this workflow.

### Quantification and statistical analysis

In general, data are represented by mean ±SD. Specific statistical tests used, p value level definitions, and additional details are listed in figure legends.

## Results & discussion

The objective of this study was to the assess the cell-damaging activities of 10 medically relevant venomous snake species from two snake families using fluorescent microscopy and a number of cellular assays commonly used for screening cytotoxic compounds. The effects of the respective species on RPTEC/TERT1 cells were evaluated with increasing venom concentrations and at varying exposure times. We subsequently combined the cellular bioassays with advanced analytical tools to characterise the compounds responsible for the observed cell-damaging activities of two distinct snake species.

### Morphological assessment of venom effects

To provide an overview of the presence and level of activity within our panel of snake venoms we collected representative brightfield, digital phase contrast and immunofluorescence microscopy images allowing us to study the morphology of the cells over time. Cells responded differently to the various types of venom, with morphological alterations of the cells depending on the snake species, the venom concentration and the duration of exposure.

**Fig 2:**
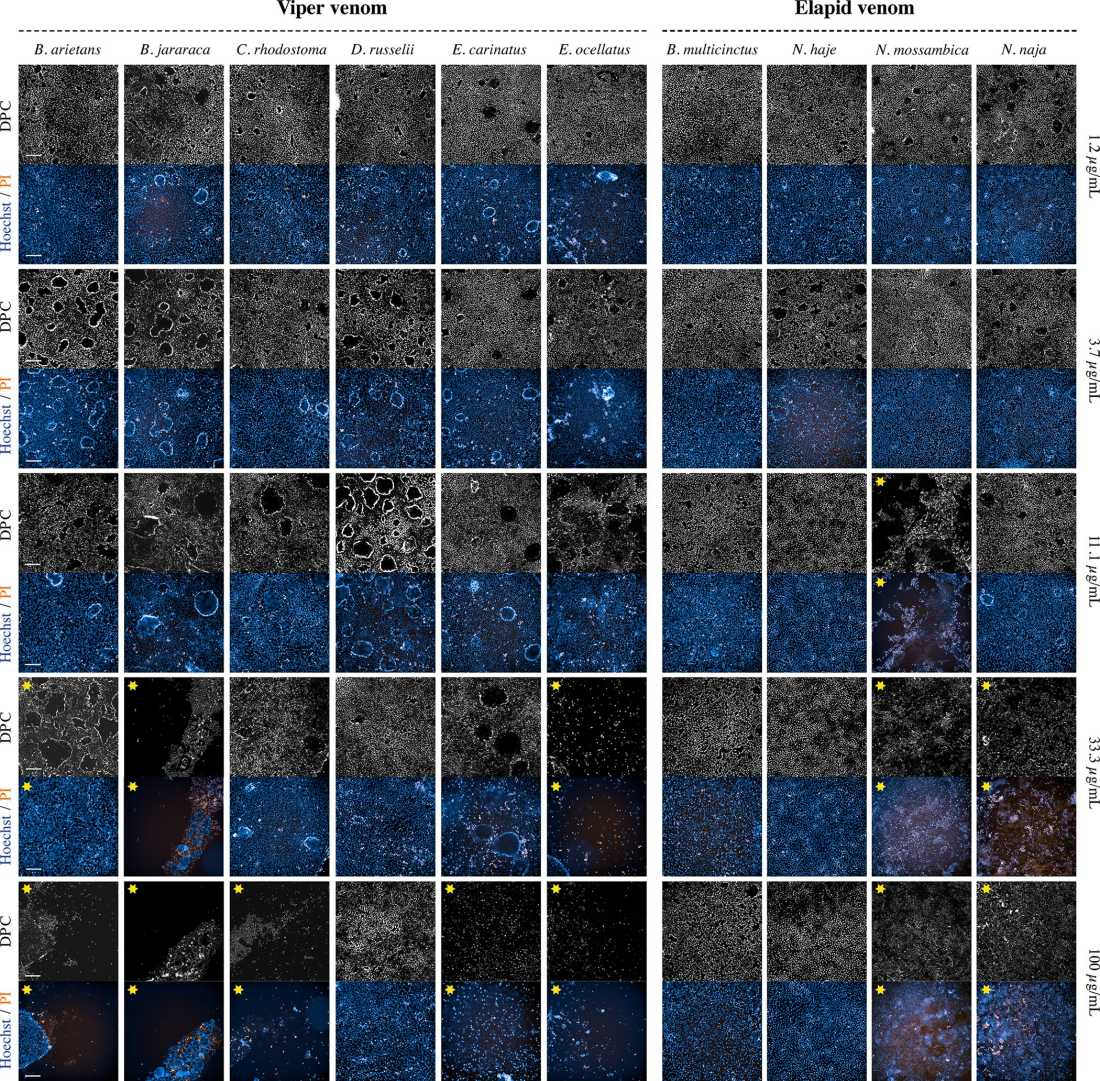
Digital phase contrast (DPC) and immunofluorescent microscopy images showing morphology of RPTEC/TERT1cells after 24 hours in presence of ten medically relevant snake species at increasing venom concentrations. Both the DPC and fluorescent images were captured using confocal microscopy with 10X magnification. H342 staining is shown in blue, and PI in orange. All exposure settings were kept the same. The scale bar represents 200 μm. 0.1% Triton T-100 was used as a positive control. The images are scaled post-acquisition to the positive control. Yellow stars represent wells in which at least some activity was observed.

**Fig 3:**
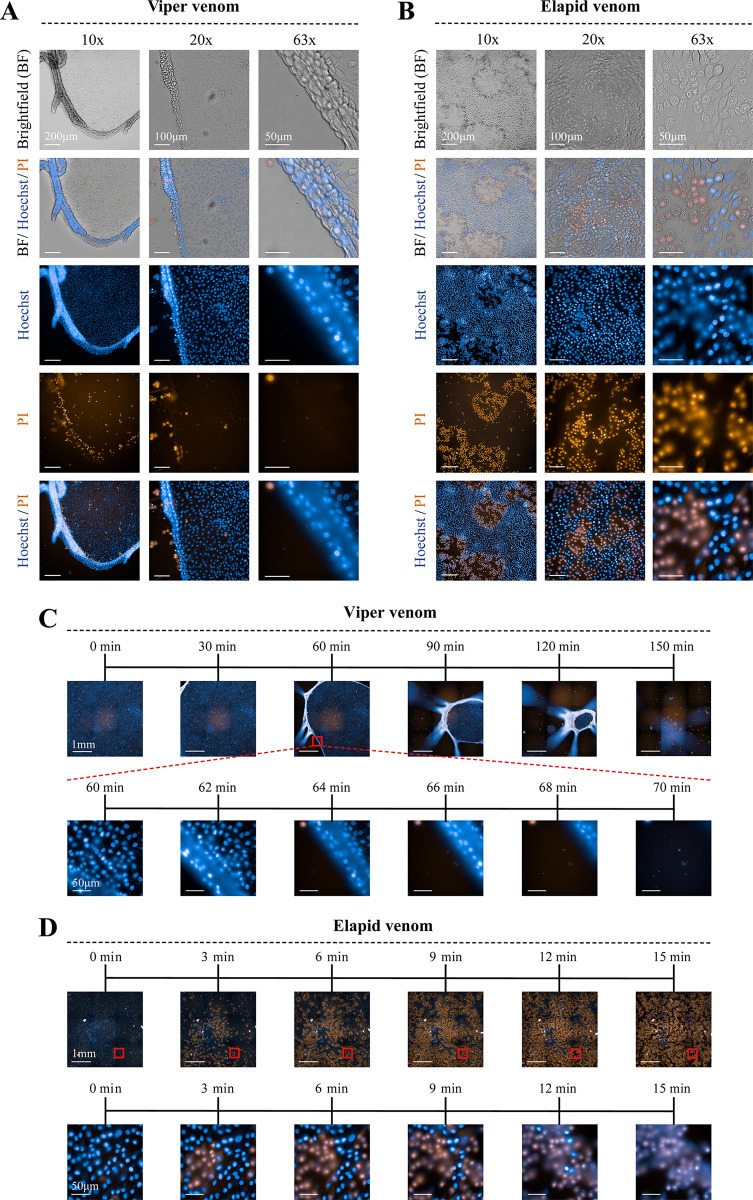
Brightfield and immunofluorescent microscopy images showing the difference in morphology of RPTEC/TERT1 cells in presence of two venoms with contrasting effects. (A) Cells exposed to 100 μg/mL *E*. *ocellatus* venom, with various channels (brightfield, Hoechst & PI) at 10X, 20X and 63X magnification. (B) Cells exposed to 100 μg/mL *N*. *mossambica* venom, with various channels (brightfield, Hoechst & PI) at 10X, 20X and 63X magnification. (C). Time series (0–150 min) of the effects of *E*. *ocellatus* venom (100 μg/mL), with a higher-magnification section (63X) clearly showing the monolayer detachment (60–70 min). (D). Time series (0–15 min) of the effects of *N*. *mossambica* venom (100 μg/mL) on cells, with a higher-magnification section (63X) showing the PI entering the cell (0–15 min). Scale bars lengths represented in the images.

After 3 hours of incubation, the effect of the venoms of *B*. *arietans*, *B*. *jararaca*, *C*. *rhodostoma*, *E*. *carinatus* and *E*. *ocellatu*s became visible. All four venoms caused detachment of the monolayer (which under normal conditions is attached to the bottom of the well). This detachment could be observed by the retraction of the monolayer, mostly from the corners inwards, after which the monolayer curled further upwards towards the middle of the well (Figs 2 and 3 and S2–S6). The level of detachment generally increased over time and with higher venom concentrations (S1–S6Figs). After 24 hours, the top concentration of all viper species, excluding *D*. *russelii*, resulted in a more or less complete detachment of the cell monolayer (Fig 2). At higher magnifications, the detachment of the monolayer was clearly visible, resembling a rolled-up sleeve (Fig 3A and 3C and S1 Movie). Time of exposure had a significant effect on the extent of monolayer detachment (Figs 3 and S1–S6). Interestingly, despite the striking effects in terms of monolayer detachment, membrane integrity was hardly affected, which could be deduced from the fact that PI was excluded from the cells, even after prolonged venom exposure (Figs 2 and S6)

The venoms of *N*. *mossambica* and *N*. *naja* affected the cells in a completely different manner, with the monolayer being largely unaffected. These venoms seemed to affect the cell membranes instead, shown by the fact that PI entered the cells, staining the inside of the cell and the cell nucleus (Fig 3B and 3D and S2 Movie). The number of PI-stained cells increased over time and with higher venom concentrations. The most striking effect was observed for 100 μg/mL of *N*. *mossambica* venom, reaching a maximum effect within the first 15 minutes post-venom addition (Fig 3D and S2 Movie). For *B*. *multicinctus* and *N*. *haje* we observed little to no effect during the first 24 hours of incubation, even at higher concentrations (Fig 2). Only after 48 hours at the highest concentration of *N*. *haje* venom an effect was observed, similar to that of *N*. *mossambica* and *N*. *naja* (S6 Fig). Although cells that were exposed to these venoms did not show any clear morphological alterations, we did observe a relatively large number of ‘domes’ in these cells compared to the negative control (Figs 2A and S2–S6). These are typical three-dimensional cellular structures in epithelial cells which are a characteristic of healthy cells.

### Quantitative assessment of venom effects

#### Live cell count (Hoechst / PI)

In order to complement the morphological data presented above we used the same chromosome stains (i.e. H342 and PI), which provided both qualitative and quantitative data.

In cells incubated with viper venoms, PI was unable to cross the cell membrane even after prolonged incubation resulting in low numbers of PI-stained cells. However, the quantitative data suggests differently. After 24 hours of incubation, all viper venoms (at 100 μg/mL) resulted in cell counts close to zero (S7B Fig). This shows that live cell counts for the viper venom data should be interpreted with caution, as the morphological data clearly shows that the cells did not become PI-permeable (Figs 2 and S2–S6). This observation will be further discussed in section ‘Matching morphological & quantitative data’.

The graphs representing the effects of *N*. *mossambica* and *N*. *naja* venoms show a potent cytotoxic effect for higher venom concentrations (S7B Fig). This is in line with the qualitative data presented above, which accordingly showed that the highest venom concentrations heavily affected the permeability of the cells towards PI (Figs 2 and S2–S6). The graphs further show that the live cell count decreases with higher concentrations in a dose-dependent manner, as is the case for longer exposure times (S7B Fig). For *B*. *multicinctus* and *N*. *haje* venoms we did not observe any notable effects for the first 24 hours of exposure. After 48 hours of incubation a decrease in the number of live cells relative to negative control for 100 μg/mL of *N*. *haje* venom could be seen (S7B Fig).

#### Cell area quantification

As discussed above, quantification of the ratio of live cells relative to the total cell count proved to be challenging for those venoms that affected the cell monolayer in such way that the total (live) cell count could not be properly determined. We therefore incorporated a method to quantify the level of activity in terms of their ability to destabilise the cell monolayer. This provided us with a useful method of assessing the level of ECM-degrading activity.

For all viper venoms except *D*. *russelii*, subtraction of the monolayer was observed, with the magnitude of activity depending on the species, the venom concentration and the time of exposure (Figs 2 and S2–S6). The graphs closely match the morphological presented above. After 6 hours of exposure to 100 μg/mL of venom of *B*. *jararaca*, *C*. *rhodostoma* and *E*. *ocellatus* we observed near to complete subtraction of the monolayer, which is represented by a cell surface area close to zero (S8A Fig). When incubated for 12–24 hours, the graphs show potent activity for all viper venoms (excluding *D*. *russelii* venom), with *B*. *jararaca* and *E*. *ocellatus* the most potent, and the top two concentrations causing complete or near to complete subtraction of the monolayers. Maximal activity was observed after 48 hours of exposure, with all five viper venoms showing complete subtraction at 33.3 μg/mL and 100 μg/mL, and with *E*. *ocellatus* causing potent monolayer destabilizing activity at 11.1 μg/mL (S8A Fig).

For *N*. *mossambica* and *N*. *naja* venoms, the graphs representing live cell count and cell surface area matched closely (S8 Fig). After 48 hours of incubation, the graphs of *N*. *mossambica* and *N*. *naja* demonstrated a significant decrease in cell surface area for the top three concentrations of *N*. *mossambica*, and the two highest concentrations of *N*. *naja*, venom. The graphs associated with the venoms of *B*. *multicinctus* and *N*. *haje* match both the morphological and live cell count data (S2–S6 and S8 Figs), with little activity observed even at higher venom concentrations and prolonged exposure.

#### Resazurin reduction

Cell metabolism was monitored using the amount of resazurin reduction as a proxy for metabolic activity and cellular stress.

After 3 hours of incubation with viper venoms, the metabolic rate in the wells with the top concentrations increased substantially, which was most notable with the venom of *E*. *carinatus* (S9A Fig). In the subsequent hours, the metabolic rates decreased to the level of the negative control, followed by a further decrease for the higher venom concentrations as the cells are incubated for over 24 hours. The only exception is *D*. *russelii*, for which the metabolic rate after an initial increase later resembles the negative control.

For *N*. *mossambica* and *N*. *naja*, a striking drop could be observed for the metabolic activity could be observed at all exposure times for the top two concentrations. A minimal increase in metabolic activity was seen for *B*. *multicinctus* and *N*. *haje* venom (S9B Fig). After 24 hours of incubation there was a slight decrease for the cells incubated with 100 μg/mL of *N*. *haje* venom. This is in line with the minor effects of these venoms on the live cell count and cell surface area data discussed above.

#### ATP assay

In order to complement the data presented above we further included cell viability in our panel of bioassays. For this, we investigated the cells that were incubated for 24 and 48 hours with the respective venoms and subsequently lysed the cells and measured ATP levels.

For all viper species except *D*. *russelii* we see lower levels of ATP relative to the negative control for the top concentrations of venom (S10A Fig) After 48 hours of exposure this decrease continued, with *B*. *jararaca* and *E*. *ocellatus* affecting cell viability at 3.7 μg/mL and 1.2 μg/mL (S10A Fig). For *D*. *russelii* we see a more subtle decrease in ATP levels for the top three concentrations, but nothing substantial.

Similar levels of cell viability decrease were observed for *N*. *mossambica* and *N*. *naja* venoms, with the former being slightly more potent (S10B Fig). After 48 hours, the viability for all five concentrations of both species has decreased, although only the top concentrations caused a steady drop in the ATP levels measured (S10B Fig). Contrastingly, *B*. *multicinctus* and *N*. *haje* venom were not capable of causing a notable difference in cell viability except for the top concentrations of *N*. *haje* venom after a 48-hour incubation period, which resulted in a subtle drop in viability (S10B Fig).

### Matching morphological & quantitative data

**Fig 4:**
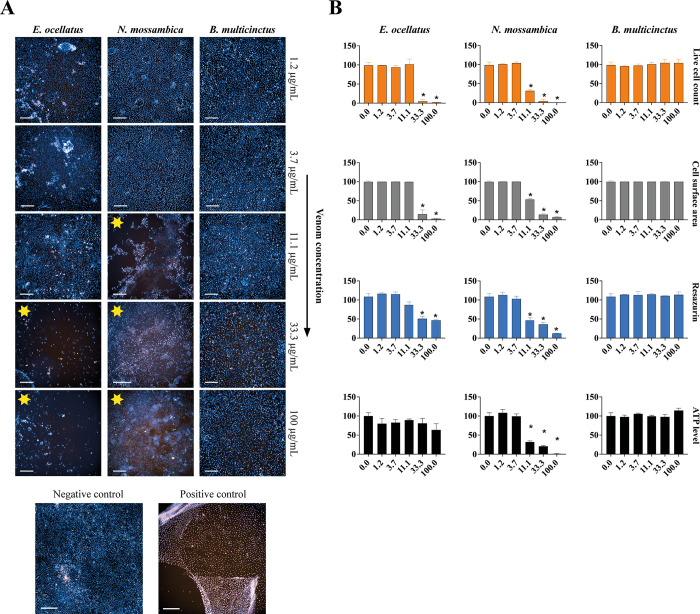
Comparison of morphological data and quantitative data for three venoms that have varying effects on the cells. (A) Morphological data represented by immunofluorescent microscopy images showing morphology of RPTEC/TERT1cells after 24 hours exposure. Images were captured using confocal microscopy with 10X magnification. H342 staining is shown in blue, and PI in orange. 0.1% Triton T-100 was used as a positive control. Yellow stars represent wells in which activity was observed. Scale bar represents 200 μm. (B) Quantitative data of four cellular bioassays shown as bar graphs, with the activity of the venom represented relative to negative control (0 μg/mL). Live cell count (orange); cell surface area (grey); resazurin reduction activity (blue); ATP level (black). Increasing venom concentrations on the X-axes (in μg/mL) and percentage relative to negative control on the Y-axes. Measurements are presented as the mean of three individual experiments (N = 3), error bars depict SD; ‘*’ represents a statistically significant difference when compared to negative control, two-tailed test, p < 0.05 (Bonferroni-corrected).

Combining the data of the various bioassays allows us to quantify the level of cell-damaging activities, regardless of the mechanism involved. When comparing the morphological and quantitative data of the most potent viper venom in our panel (i.e. *E*. *ocellatus*), we see that the graphs representing live cell count and cell area match closely (Fig 4A). As discussed, this observation suggests that the live cell counts for vipers venoms should be interpreted with caution, as the morphological data clearly shows that the cells did not become PI-permeable. An explanation for this could be the fact that the detachment of the monolayer caused the cells to move out of the focus of the microscope, hindering the cell count. Other methods were therefore needed to properly quantify the cell-damaging properties of the viper venoms. Calculation of the cell surface area provided a more suitable way of quantifying the activity of viper venoms, which could be easily calculated with any microscope with camera mount and the appropriate software. The observed viper venom effects were less obvious in the graphs for resazurin reduction compared to the graphs for live cells count and cell surface area (Figs 4 and S7–S9). Also, an initial increase in resazurin metabolism was be observed after 3 hours of incubation for higher concentrations of viper venom. As this assay is a measure for active metabolism and resazurin is generally postulated as being reduced by mitochondrial enzymes, the increase in metabolism could possibly be the result of mitochondrial stress in response to the presence of the venom [34–37]. As these observations suggest that venom addition may have caused an increase in metabolic activity it was decided to also include the ATP-assay as a direct measure of cell viability [38,39]. No proper trend could be observed for the ATP-content in the lysates, which suggests that cell viability was not radically altered by the viper venoms, as ATP-levels tend to decline rapidly when cells undergo necrosis or apoptosis [38,39]. This observation is in line with the fact that for the first ~24 hours, except for detachment of the monolayer, the cells were not affected. Only after prolonged incubation did the cells start to exhibit significant loss in viability (Figs 3 and S7). For the elapid venoms, the graphs show similar trends for all bioassays, regardless of the level of bioactivity (Figs 4B and S7–S10).

### Identification of bioactive compounds in fractionated venoms

To characterise the venom components responsible for the observed cell-damaging activity, we correlated the bioactivity peaks with proteomics data in order to improve the toxin identification obtained with the initial correlation analyses. The most potent venom of each snake family was selected for further analysis using a similar nanofractionation approach as described by Slagboom et al. [33]. This was done in an attempt to identify the toxins responsible for the observed affect and to demonstrate the complete analytical workflow.

#### Correlation of bioactivity peaks with proteomics data

Venoms of *N*. *mossambica* and *E*. *ocellatus* at different concentrations (i.e. 0.5, 1.0 and 2.5 mg/mL) were fractionated using RP-HPLC. For all (bioactive) fractions of each species the corresponding bioactivity chromatogram and proteomics data was acquired. For the bioassay chromatograms, the average Y-axis signal at the beginning of each chromatogram represents the baseline, (i.e. ‘normal’ cell activity), whereas the negative peaks (dips) correspond to the bioactive fractions in the wells. In order to try and improve the toxin identification obtained with the initial correlation, we performed tryptic digestion on the venom fractions, after which the proteomics data was correlated to the bioactivity data.

#### Identification of membrane-disrupting toxins in *N*. *mossambica* venom

The venoms of some of the elapids, including *N*. *mossambica* were capable of affecting the membrane integrity, which was shown by the fact that PI could enter the cell (Fig 3). As cell membrane integrity is crucial for the normal functioning of the cell, it is not surprising that certain toxin families act specifically on the cell membrane [7,8,40]. Destabilisation of cellular membranes can be caused by cytotoxic 3FTxs, PLA_2_s and ß-defensin like toxins, having different mechanisms of action [7,8].

**Fig 5:**
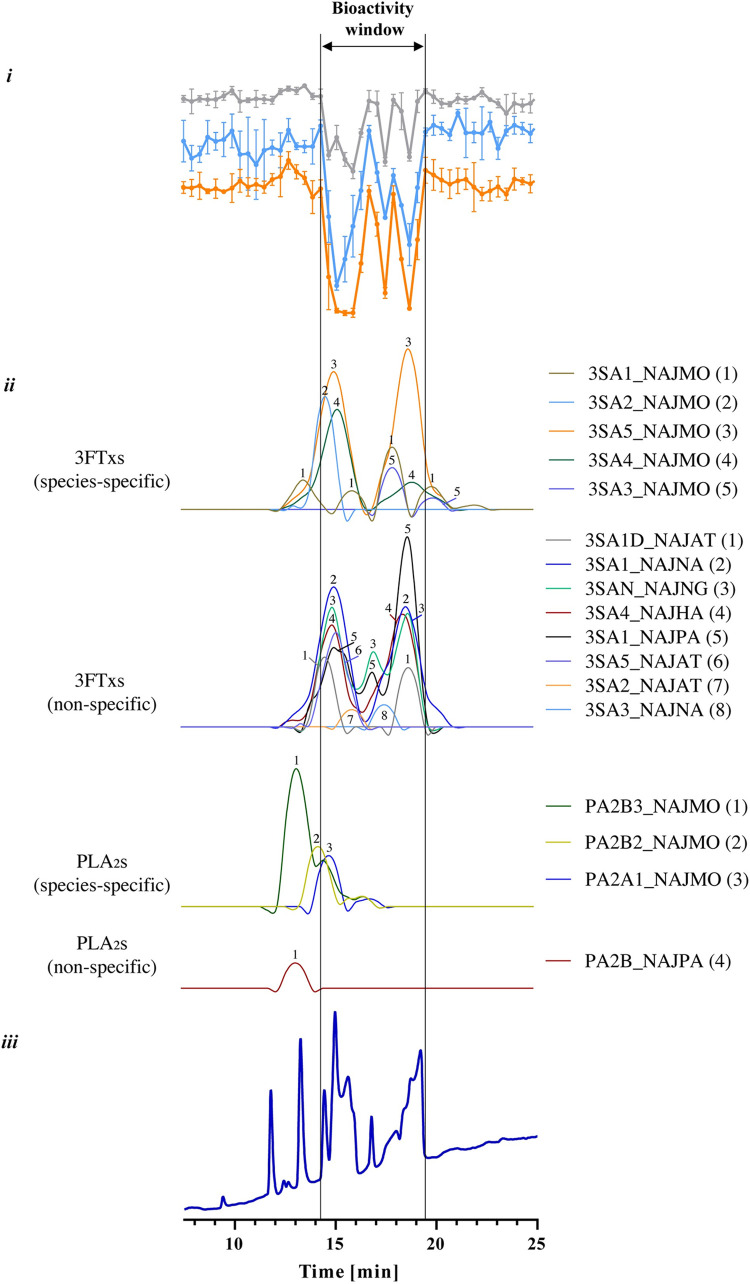
Identification of bioactive fractions of *N*. *mossambica* venom (1 mg/mL) by correlating bioactivity data with proteomics data. i: bioactivity chromatograms obtained by plotting the results of three bioassays: cell surface area (grey); resazurin reduction (blue); live cell count (orange). The peaks with negative minima indicate the presence of bioactive fractions; ii: Graphs representing the protein score chromatograms (PSCs), showing the individual venom proteins found with Mascot database searching of the digested well fractions. iii: UV traces of the snake venoms at 220 nm obtained by RP-HPLC. The vertical outer lines mark the bioactivity window, which includes the main activity peaks and their corresponding PSC peaks and RP-HPLC-UV chromatogram peaks. Measurements are presented as the mean of three individual experiments (N = 3), error bars depict SD.

For *N*. *mossambica*, activity was observed in a dose-dependent manner for all assays. At a concentration of 1.0 mg/mL, three bioactivity peaks were present between 14.2–19.4 min (Fig 5). Within this bioactivity window, a total of 17 toxins could be identified using proteomics. Of these, 13 toxins were found to be part of the 3FTx toxin family and three to the PLA_2_ family of toxins (Fig 5 and S1 Table). Assigning toxins to specific bioassay peaks was difficult, as for some toxins (e.g. 3SA5_NAJMO; 3SA1_NAJNA; 3SA4_NAJHA) there are multiple peaks present. Previous studies have argued that these peaks could be different toxins with sequence similarities that are not yet in the database and are therefore assigned to their closest homologue, as was described by Slagboom et al. [27,41]. According to the Uniprot database the 3FTx proteins identified in this study are ‘showing cytolytic activity on many different cells by forming pores in lipid membranes’ [42–48]. 3FTxs perform a variety of biological functions, including neuro-, haemo- and cytotoxicity which are caused by a variety of mechanisms [16,17,49–54] The exact mechanism by which these toxins execute their damaging effects on cells remains unclear, with competing hypotheses present, including pore formation and interactions with cell-surface proteins [17,55–60]. The venoms of *N*. *mossambica* and *N*. *naja* are particularly rich in 3FTx, with relative abundances ranging from 63.8–80.5% (S2 Table). Other toxins that were found within the bioactivity window were the PLA_2_s, which described in Uniprot as ‘showing several activities, including (in)direct haemolytic action, myonecrosis and strong anticoagulant activity [61–63]. PLA_2_s disrupt the plasma membrane by hydrolysing membrane phospholipids, resulting in the alteration of the membrane structure, whereas enzymatically inactive PLA_2_s may act via direct perturbation of the membrane [13,64–67].

#### Identification of toxins causing monolayer detachment in *E*. *ocellatus* venom

Incubation of cells with the venoms from all vipers (except *D*. *russelii*) resulted in the detachment and subsequent subtraction of the cell monolayer without affecting the cells directly (Figs 2 and 4). This is further supported by the fact that cell viability and cell metabolism were less affected then observed for the venoms of *N*. *mossambica* and *N*. *naja*. This suggests that the viper venoms might affect the ECM, which is a macromolecular scaffold which attaches the cell monolayer to the bottom of the wells. The main components of the ECM are proteoglycans, glycoproteins and fibrous proteins such as collagen, elastin, fibronectin and laminin [11,40,68,69]. Certain toxin classes such as SVMPs and hyaluronidases are known to proteolytically degrade ECM components [1,11,25,70–72].

**Fig 6:**
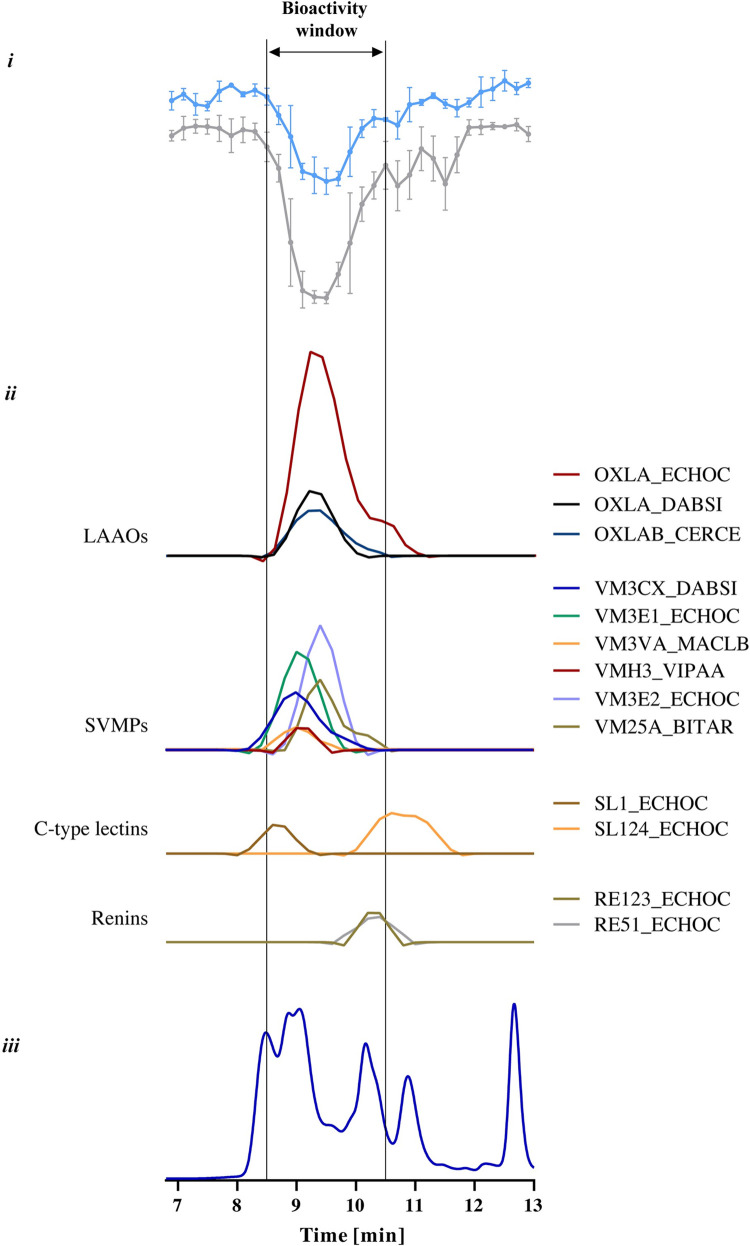
Identification of bioactive fractions of *E*. *ocellatus* venom (5 mg/mL) by correlating bioactivity data with proteomics data. i: bioactivity chromatograms obtained by plotting the results of two assays: resazurin reduction (blue); cell surface area (grey). The live cell count was not included as the graphs do not accurately describe the number of live cells as a result of the detachment of the monolayer (see also above). The peaks with negative minima indicate the presence of bioactive fractions; ii: Graphs representing the protein score chromatograms (PSCs), showing the individual venom proteins found with Mascot database searching of the digested well fractions; iii: UV traces of the snake venoms at 220 nm obtained by SEC-HPLC. The vertical outer lines mark the bioactivity window, which includes the main activity peaks and their corresponding PSC peaks and SEC-UV chromatogram peaks. Measurements are presented as the mean of three individual experiments (N = 3), error bars depict SD.

Surprisingly, the separated fractions of *E*. *ocellatus* did not show any bioactivity on neither of the assays even at higher concentrations (S11B Fig). A possible explanation could be the fact that the responsible toxins are subject to denaturation as a result of the solvent conditions present during RP-HPLC separation. This is in line with previous studies on the effects of organic modifiers and acidifiers on protein stability [73–75]. In order to address this issue, we decided to use SEC-HPLC as an alternative separation method, which uses an eluent based on non-volatile salt buffers with DPBS as the mobile phase. The use of SEC allowed us to separate and collect the venom fractions in their native form. We then subjected the venom fractions to the same bioassays discussed above. The results demonstrate that the venom fractions separated using a non-volatile buffer retained their activities, showing effects similar to what was observed in the crude venoms (Figs 6 and S12).

Fractions of *E*. *ocellatus* venom eluted between 8 and 13 min, with at least six peaks and a number of co-eluting peaks that can be observed in the SEC-UV chromatogram (Fig 6). Bioactive fractions eluted from approx. 8.5 to 10.5 min. A total of 13 toxins could be identified, belonging to the following toxin classes: LAAOs (three toxins), SVMPs (six toxins), C-type lectins (two toxins) and renins (two toxins) (Fig 6 and S3 Table). The LAAOs that were found (i.e. OXLA_ ECHOC, OXLA_ DABSI and OXLAB_ CERCA) belong to a family of toxins that is thought to be associated with the induction of oxidative stress which may induce cell damage. These toxins may exhibit a diverse array of biological activities, including haemorrhage, haemolysis and apoptosis of various cell types [21,76–80]. The SVMPs that were identified within the bioactivity window have been shown to be proteolytically active and to exhibit strong haemorrhagic activity [81–83]. In addition to LAAO and SVMPs, we found two C-type lectins (SL1_ ECHOC; SL124_ECHOC) in the bioactive fractions, a family of toxins for which a diverse range of effects is described (including cytotoxicity), although the exact mechanisms have not been elucidated [84–87]. The other toxins represented in the graphs fall outside of the bioactivity window and are not known to exhibit proteolytic activity [1,7].

### Concluding remarks

This study describes the development of a medium throughput approach for the identification of venom components associated with cell- and tissue damaging effects. We used a set of 10 medically relevant species, which were screened on a panel of cellular bioassays. The results of this study revealed a notable difference in the way in which the venoms of elapids and vipers exert their effects on RPTEC/TERT1 cells. The venoms of *N*. *mossambica* and *N*. *naja* affected the integrity of the cell membrane (showed by the fact that PI was able to cross the cell membrane within minutes after incubation. The responsible toxins could therefore be considered ‘true’ cytotoxins, as these directly affected the cells by disrupting cell membrane integrity. Viper venoms on the contrary affect the substrates of the ECM-scaffold, probably by proteolytic degradation of the matrix substrates. This resulted in the detachment of the cell monolayer, which is a secondary effect of degradation of the ECM. The RPTEC/TERT1 cell monolayer provided a suitable model for differentiating between the two distinct cell-damaging mechanisms of action, namely membrane-disruption and ECM-degradation. Although kidney cells used in this study might have different physiological properties as opposed to other cell types (e.g. endothelial cells), these cells can be used as a model to study cell-damaging effect of snake venoms. The combination of RP-HPLC and SEC-HPLC, post-column cellular bioassaying and proteomics allows for the parallel identification of membrane-disrupting and ECM-degrading venom components. The application of this approach could be valuable for better understanding venom-induced cell- and tissue damage and could be used in the future development of snakebite therapeutics targeting (local) tissue damage caused by envenoming.

## Supporting information

S1 Methods **Additional Methods, Section 1.** Elaboration of the Methods section of the manuscript, explaining in more detail the section: “Liquid chromatography, nanofractionation and mass spectrometry”.**Additional Methods, Section 2.** Elaboration of the Methods section of the manuscript, explaining in more detail the section: “Obtaining bioassays chromatograms after fractionation with SEC-HPLC”. **Additional Methods, Section 3.** Elaboration of the Methods section of the manuscript, explaining in more detail the section: “Identification of bioactive fractions by post-column proteomics”.
(DOCX)

S1 Table Toxins identified by nano-LC-MS/MS following tryptic digestion of fractionated toxins from the venom of *N*. *mossambica*. Abbreviations: prot_acc, protein accession number; prot_score, protein score; prot_cover, protein coverage; prot_desc, protein description; prot_seq, protein sequence; pep_seq, peptide sequence.
(XLSX)

S2 Table Overview of the 10 species included in the study with the proportion of the 12 major protein families in each venom (as percent of total venom). Abbreviations: PLA_2_, phospholipase A2; SVSP, snake venom serine protease; SVMP, snake venom metalloprotease; LAAO, L-amino acid oxidase; 3FTx, three-finger toxin; KUN, Kunitz peptides; CRiSP, Cysteine-Rich Secretory Protein; NP, natriuretic peptide; %WV, percentage of venom.
(DOCX)

S3 Table Toxins identified by nano-LC-MS/MS following tryptic digestion of fractionated toxins from the venom of *E*. *ocellatus*. Abbreviations: prot_acc, protein accession number; prot_score, protein score; prot_cover, protein coverage; prot_desc, protein description; prot_seq, protein sequence; pep_seq, peptide sequence.
(XLSX)

S1 Fig Brightfield (BF) and immunofluorescent microscopy images showing morphology of RPTEC/TERT1cells in absence of venoms (0 hours). Both BF and fluorescent images were captured using confocal microscopy with 10X magnification. H342 staining is shown in blue, and PI in orange. All exposure settings were kept the same. The scale bar represents 500 μm. The images were scaled post-acquisition to the positive control.
(TIF)

S2 Fig Brightfield (BF) and immunofluorescent microscopy images showing morphology of RPTEC/TERT1cells after 3 hours in presence of ten medically relevant snake species at increasing venom concentrations. Both BF and fluorescent images were captured using confocal microscopy with 10X magnification. H342 staining is shown in blue, and PI in orange. All exposure settings were kept the same. The scale bar represents 500 μm. The images were scaled post-acquisition to the positive control. Yellow stars represent wells in which at least some activity was observed.
(TIF)

S3 Fig Brightfield (BF) and immunofluorescent microscopy images showing morphology of RPTEC/TERT1cells after 6 hours in presence of ten medically relevant snake species at increasing venom concentrations. Both BF and fluorescent images were captured using confocal microscopy with 10X magnification. H342 staining is shown in blue, and PI in orange. All exposure settings were kept the same. The scale bar represents 500 μm. The images were scaled post-acquisition to the positive control. Yellow stars represent wells in which at least some activity was observed.
(TIF)

S4 Fig Brightfield (BF) and immunofluorescent microscopy images showing morphology of RPTEC/TERT1cells after 12 hours in presence of ten medically relevant snake species at increasing venom concentrations. Both BF and fluorescent images were captured using confocal microscopy with 10X magnification. H342 staining is shown in blue, and PI in orange. All exposure settings were kept the same. The scale bar represents 500 μm. The images were scaled post-acquisition to the positive control. Yellow stars represent wells in which at least some activity was observed.
(TIF)

S5 Fig Brightfield (BF) and immunofluorescent microscopy images showing morphology of RPTEC/TERT1cells after 24 hours in presence of ten medically relevant snake species at increasing venom concentrations. Both BF and fluorescent images were captured using confocal microscopy with 10X magnification. H342 staining is shown in blue, and PI in orange. All exposure settings were kept the same. The scale bar represents 500 μm. The images were scaled post-acquisition to the positive control. Yellow stars represent wells in which at least some activity was observed; red arrows depict areas where monolayer the start of monolayer detachment can be observed.
(TIF)

S6 Fig Brightfield (BF) and immunofluorescent microscopy images showing morphology of RPTEC/TERT1cells after 48 hours in presence of ten medically relevant snake species at increasing venom concentrations. Both BF and fluorescent images were captured using confocal microscopy with 10X magnification. H342 staining is shown in blue, and PI in orange. All exposure settings were kept the same. The scale bar represents 500 μm. The images were scaled post-acquisition to the positive control. Yellow stars represent wells in which at least some activity was observed.
(TIF)

S7 Fig Bar graphs representing the time series data for the live cell count in response to our panel of 10 snake species relative to the negative control (A) Time series data (3; 6; 12; 24; 48 hours) of six viper species at increasing venom concentrations (0; 1.2; 3.7; 11.1; 33.3; 100 μg/mL). (B) Time series data (3; 6; 12; 24; 48 hours) of four elapid species at increasing venom concentrations (0; 1.2; 3.7; 11.1; 33.3; 100 μg/mL). Venom concentrations on the X-axes (in μg/mL) and percentage relative to negative control on the Y-axes. Measurements are presented as the mean of three individual experiments (N = 3), error bars depict SD.
(EPS)

S8 Fig Bar graphs representing the time series data for the cell surface area in response to our panel of 10 snake species relative to the negative control (A) Time series data (3; 6; 12; 24; 48 hours) of six viper species at increasing venom concentrations (0; 1.2; 3.7; 11.1; 33.3; 100 μg/mL). (B) Time series data (3; 6; 12; 24; 48 hours) of four elapid species at increasing venom concentrations (0; 1.2; 3.7; 11.1; 33.3; 100 μg/mL). Venom concentrations on the X-axes (in μg/mL) and percentage relative to negative control on the Y-axes. Measurements are presented as the mean of three individual experiments (N = 3), error bars depict SD.
(EPS)

S9 Fig Bar graphs representing the time series data for the resazurin reduction in response to our panel of 10 snake species relative to the negative control (A) Time series data (3; 6; 12; 24; 48 hours) of six viper species at increasing venom concentrations (0; 1.2; 3.7; 11.1; 33.3; 100 μg/mL). (B) Time series data (3; 6; 12; 24; 48 hours) of four elapid species at increasing venom concentrations (0; 1.2; 3.7; 11.1; 33.3; 100 μg/mL). Venom concentrations on the X-axes (in μg/mL) and percentage relative to negative control on the Y-axes. Measurements are presented as the mean of three individual experiments (N = 3), error bars depict SD.
(EPS)

S10 Fig Bar graphs representing the time series data for the ATP concentration in response to our panel of 10 snake species relative to the negative control (A) Time series data (3; 6; 12; 24; 48 hours) of six viper species at increasing venom concentrations (0; 1.2; 3.7; 11.1; 33.3; 100 μg/mL). (B) Time series data (3; 6; 12; 24; 48 hours) of four elapid species at increasing venom concentrations (0; 1.2; 3.7; 11.1; 33.3; 100 μg/mL). Venom concentrations on the X-axes (in μg/mL) and percentage relative to negative control on the Y-axes. Measurements are presented as the mean of three individual experiments (N = 3), error bars depict SD.
(EPS)

S11 Fig Bioactivity profiles of *N. mossambica* and *E. ocellatus* at various concentrations (0.5; 1; 2.5 mg/mL) after fractionation with RP-HPLC. Bioactivity chromatograms obtained by plotting the results of three assays: live cell count (orange); cell surface area (grey); resazurin reduction (blue). The peaks with negative minima indicate the presence of bioactive compounds. Red dots represent the positive control, green dots represent the negative control. Time (in min) is represented on the X-axes and percentage relative to negative control is given on the Y-axes. Measurements are presented as the mean of three individual experiments (N = 3), error bars depict SD. Measurements are presented as the mean of three individual experiments (N = 3), error bars depict SD.
(EPS)

S12 Fig Bioactivity profiles of *E*. *ocellatus* at two concentrations (2.5; 5.0 mg/mL) after fractionation with SEC. (A) Bioactivity profiles for venom concentrations 5.0 mg/mL and 2.5 mg/mL that were presented to the cells in an 80/20% ratio (i.e. 80% venom fractions combined with 20% growth medium. (B) Bioactivity profiles of venom (2.5 mg/mL) that was given to the cells in an 50/50% ratio. Bioactivity chromatograms obtained by plotting the results of two assays: live cell count (orange) and cell surface area (grey) The peaks with negative minima indicate the presence of bioactive compounds respectively. Time (in min) is represented on the X-axes and percentage relative to negative control is given on the Y-axes. Measurements are presented as the mean of three individual experiments (N = 3), error bars depict SD. Data of the 5 mg/mL fractions are presented as the mean of three individual experiments (N = 3), error bars depict SD; measurements of the bioactivity profiles for venom concentrations of 2.5 mg/mL are N = 1.
(TIF)

S13 Fig Immunofluorescent microscopy images showing the staining of RPTEC/TERT1 cells with H342 and PI, which were used to quantify the total cell count. Conditions shown for three different venoms: cytotoxic viper, cytotoxic elapid and non-cytotoxic elapid. (A) Image of cells stained with H342 (blue) and PI (orange) in the top row, with the lower set of images demonstrating the ability of the software to calculate all H342-stained cells (coloured dots). (B) Image of cells stained with PI alone, with the lower row showing the PI-stained cells as recognised by the software. Scale bar represents 200 μm.
(EPS)

S14 Fig Brightfield microscopy images of RPTEC/TERT1 cells in response to three different venoms, which were used to quantify the cell surface area. Conditions shown for three different venoms: cytotoxic viper, cytotoxic elapid and non-cytotoxic elapid. Top row represents the ‘training’ of the software to differentiate cell populations (red dots) from the ‘empty well area’ (green dots) using a simple learn-by-example approach. The lower set of images provides the calculated cell surface area (depicted in red) and the ‘empty well area’ (in green). The scale bar represents 200 μm.
(EPS)

S1 Movie Microscopic video showing RPTEC/TERT1 cells in response to *E*. *ocellatus* venom. Time series (30–60 min) of the effects of *E*. *ocellatus* venom (100 μg/mL) on cells at a magnification of (63X magnification), clearly showing the monolayer detachment. The video is composed of individual images taken with the high content imager, with 2 min steps between each individual image (15 images total), image rate: 1fps.
(MP4)

S2 Movie Microscopic video showing RPTEC/TERT1 cells in response to *N*. *mossambica* venom. Time series (0–15 min) of the effects of *N*. *mossambica* venom (100 μg/mL) on cells at a magnification of (63X magnification), clearly showing PI entering the cell. The video is composed of individual images taken with the high content imager, with 1 min steps between each individual image (15 images total), image rate: 1fps.
(MP4)

S1 Raw Data All raw data files, including raw bioassay data and proteomics data. (RAR)
