# Supplementary material for: Development of a high-throughput in vitro screening method for the assessment of cell-damaging activities of snake venoms
Source: PLoS Negl Trop Dis. 2023 Aug 17;17(8):e0011564. doi: 10.1371/journal.pntd.0011564 (PMC10465002; doi:10.1371/journal.pntd.0011564)
Supplement: S1 Methods — Additional Methods, Section 1. Elaboration of the Methods section of the manuscript, explaining in more detail the section: “Liquid chromatography, nanofractionation and mass spectrometry”.Additional Methods, Section 2. Elaboration of the Methods section of the manuscript, explaining in more detail the section: “Obtaining bioassays chromatograms after fractionation with SEC-HPLC”. Additional Methods, Section 3. Elaboration of the Methods section of the manuscript, explaining in more detail the section: “Identification of bioactive fractions by post-column proteomics”. (DOCX) [file pntd.0011564.s001.docx]

Section 1. Liquid chromatography and nanofractionation

For subsequent analysis of the fractions, we selected one representative species of each snake family. Three concentrations of crude venom were used for separation onto the 96-wells plates: 0.5; 1.0; 2.5 mg/mL. These plates were then subjected to the same panel of cytotoxicity assays as discussed in the main article. Crude venoms were separated by RP-HPLC, followed by parallel nanofractionation and subsequent mass spectrometry (MS) analysis. For LC separation, a Shimadzu UPLC system was used. All settings of the system were controlled with Shimadzu Lab Solutions software. 50 μL of each venom sample was injected by a Shimadzu SIL-30AC autosampler, and the two Shimadzu LC-30AD pumps were set to a total flow rate of 500 μL/min. A 150x4.6 mm Waters Xbridge Peptide BEH300 C18 analytical column with a 3.5-μm particle size and a 300-Å pore size was used for separation of the venoms. The separations were performed at 30°C in a Shimadzu CTD-30A column oven. Mobile phase A consisted of 98% H_2_O, 2% acetonitrile (ACN) and 0.1% formic acid (FA), and mobile phase B consisted of 98% ACN, 2% H_2_O and 0.1% FA. A linear increase of mobile phase B from 0% to 50% in 20 min was followed by a linear increase from 50% to 90% B in 4 min and a 5 min isocratic elution at 90% B. The starting conditions (5% B) were then reached linearly in 1 min and the column was equilibrated for 10 min at 5% B. LC fractions (1 fraction per 24 sec) were collected row by row in serpentine-like fashion on clear 96-well plates using FractioMator software. Each chromatographic run was collected into all wells of the 96-well plate. After fractionation, the plates were evaporated overnight for approximately 16 h using a Christ Rotational Vacuum Concentrator RVC 2-33 CD plus (Salm en Kipp). The plates were then stored at –20 °C until further use. Upon use, the venom fractions were diluted in 100 μL of growth medium and incubated for 20 min before transferring the venom-growth medium mixture to the corresponding wells of a 96-well cell plate.

**Section 2. Obtaining bioassays chromatograms after fractionation with SEC-HPLC**

For analysis of *E. ocellatus* venom, for which the toxin activity was negatively affected by the use of organic solvents and acidifiers used in our ‘traditional’ separation method we used an alternative approach, which enabled us to collect the venom toxins in their native form. For this, we used a combination of methods recently described by Slagboom et al. and Wachtel et al. [1,2]. In this approach SEC-chromatography was used, with an eluent based on non-volatile salt buffers with DPBS as the mobile phase. Two concentrations of *E. ocellatus* venom (i.e. 2.5 and 5.0 mg/mL) were fractionated onto 96-well plates using SEC on a Shimadzu HPLC system controlled with the Shimadzu Lab Solutions software. The venom samples were injected with a Shimadzu SIL 20AC Prominence autosampler utilizing a 20 µL injection volume. Subsequent separation was performed on a Sepax Zenix SEC-300 column (300 Å, 5 µm, 4.6 mm× 300 mm) in a Shimadzu CTO-10AC VP column oven set to 30°C. The mobile phase consisted of 100% DPBS (GibcoTM Dulbecco’s phosphate-buffered saline (no calcium, no magnesium, pH 7.0 to 7.3). using an isocratic elution over 20 min and a flow rate of 0.35mL/min. The elution of the toxins was monitored using a Shimadzu SPD-20A Prominence UV/Vis detector set at 220 and 280 nm. Between each run, the system was flushed using a solvent loop that was added to the pump system (with a mixture of 20% 2-propanol and 80% mQ), which was used between each run to flush the system and ensure proper toxin separation. Following the SEC column, the samples were collected as 12 sec fractions in a 96-well flat bottom plate (Greiner Bio-One) for 18 min, using a 6 min delay after the start of each subsequent run. For fractionation, we used a Gilson ASTED-XL autosampler rebuilt as a fraction collector, which was controlled by Ariadne software (in-house written software, v1.08j). This resulted in the collection of 60 wells, each with ~60 μL collected sample fractions. The plates were kept at 4 ◦C until use. Upon use, we transferred the venom fractions (in DPBS) to a separate 96-well plate, after which these were supplemented to a total volume of 100 μL (in a 80% (venom fractions in PBS) to 20 % (growth medium) ratio. We chose this ratio as lower ratios showed only limited activity (S8b)

**Section 3. Identification of bioactive fractions by post-column proteomics**

Following the identification of wells containing bioactive compounds, venom fractions were subjected to tryptic digestion in order to identify bioactive fractions using proteomics. For this, we used a method recently described by Slagboom et al. (2023) [1,2].

*3.1 Tryptic digestion and nanoLC-MS/MS*

After the 96-well plate was freeze-dried, 50 μL of reducing agent (0.05% β-mercaptoethanol in 25 mM ammonium bicarbonate, pH 8.2) was added to each of the selected wells followed by a 15-min incubation at 95 °C. Then, the samples were cooled to room temperature and centrifuged at 150 RCF for 10 s in a Himac CT 15RE centrifuge. Next, 20 μL of alkylating agent (12.5 mM iodoacetamide) was added and the samples were allowed to incubate in the dark at room temperature for 30 min. Subsequently, 20 μL of 0.01 μg/μL trypsin was added to each sample, followed by overnight incubation at 37 °C. The following day, 10 μL of 1.25% FA was added to quench the digestion. Finally, the plates were analysed using nanoLC-MS/MS (or stored at -20 °C until analysis). For the SEC-separated samples, we used the same protocol as described above, the only adjustment being the fact that instead of directly adding the reagents to the freeze-dried plate, we first pipetted 10 μL of each of the fractions to a separate 96-well plate.

For nanoLC separation of the tryptic digests an UltiMate 3000 RSLCnano system (Thermo Fisher Scientific) was used. The autosampler was run in partial-loop injection mode and allowed direct sampling from 96-well plates. The injection volume was set to 10 µL and injection was followed by separation on an Acclaim™ PepMap™ 100 C18 HPLC Column (150 mm x 75 µm,) with a particle size of 2 µm and a pore size of 100-Å in combination with an Acclaim™ PepMap™ 100 C18 trapping column (5 mm x 0.3 mm), with a particle size of 5 µm and a pore size of 100-Å, obtained from ThermoFisher Scientific. The mobile phase comprised of eluent A (98% water, 2% ACN, 0.1% FA) and eluent B (98% ACN, 2% water, 0.1% FA). The gradient used for the separation was as follows: 2 min isocratic separation at 5% B, linear increase to 80% B in 15 min, 3 min isocratic separation at 80% B, down to 5% B in 0.5 min and equilibration for 9 min. The column was kept at 30 °C in the column oven. Absorbance detection was performed at 254 nm followed by mass detection using a maXis Q-TOF mass spectrometer (Bruker) carrying a Bruker Captive spray source that operates in positive-ion mode. Source parameters were set as follows: source temperature: 150 °C; capillary voltage: 1.6 kV; dry gas flow: 3.0 L/min; nanoBooster pressure: 0.20 Bar. The spectral data was recorded at 2 Hz (in a 50 to 3000 m/z range). MS/MS spectra were collected using collision induced dissociation (CID) in data-dependent mode which uses 10-eV collision energy.

*3.2 Correlating bioactivity peaks with proteomics data*

Using the data obtained for the tryptic digests of the samples, protein identification was carried out using MASCOT (Matrix Science, London, United Kingdom) searches against Swiss-Prot, NCBInr and species-specific databases. The latter were generated from previously published transcriptomic data for those species that were available and were used for the identification of venom toxins for which cytotoxic activity was observed. The following search parameters were used: ESI-QUAD-TOF as the instrument type, semiTrypsin as the digestion enzyme allowing for one missed cleavage, carbamidomethyl on cysteine as a fixed modification, amidation (protein C-terminus) and oxidation on methionine as variable modifications, ± 0.05 Da fragment mass tolerance and ± 0.2 Da peptide mass tolerance. The Uniprot database generated a high number of Mascot identities from which a selection was made based on a protein score above 100. When performing the Mascot database searches, we further noticed that the search output provided a relative high number of other Mascot identities within the time range of the bioactivity peaks in the chromatogram, which are most likely resulting from other closely co-eluting low abundance toxins (deduced from their lower protein scores) [1,3]. So-called Protein Score Chromatograms (PSCs) were plotted for each identified toxin using an in-house written script. To obtain the PSCs, protein scores from each of the venom fractions were plotted against the corresponding retention times and bioactivity chromatograms, as has been described by Slagboom et al. and Wachtel et al. [1,2]

References

1. Slagboom J, Derks RJE, Sadighi R, Somsen GW, Ulens C, Casewell NR, et al. High-Throughput Venomics. J Proteome Res. 2023;22: 1734–1746. doi:10.1021/acs.jproteome.2c00780

2. Wachtel E, Bittenbinder MA, van de Velde B, Slagboom J, de Monts de Savasse A, Alonso LL, et al. Application of an Extracellular Matrix-Mimicking Fluorescent Polymer for the Detection of Proteolytic Venom Toxins. Toxins (Basel). 2023;15. doi:10.3390/toxins15040294

3. Slagboom J, Kaal C, Arrahman A, Vonk FJ, Somsen GW, Calvete JJ, et al. Analytical strategies in venomics. Microchem J. 2022;175: 107187. doi:10.1016/J.MICROC.2022.107187
