# Supplementary material for: Development of a high-throughput in vitro screening method for the assessment of cell-damaging activities of snake venoms
Source: PLoS Negl Trop Dis. 2023 Aug 17;17(8):e0011564. doi: 10.1371/journal.pntd.0011564 (PMC10465002; doi:10.1371/journal.pntd.0011564)
Supplement: S2 Table — Abbreviations: PLA2, phospholipase A2; SVSP, snake venom serine protease; SVMP, snake venom metalloprotease; LAAO, L-amino acid oxidase; 3FTx, three-finger toxin; KUN, Kunitz peptides; CRiSP, Cysteine-Rich Secretory Protein; NP, natriuretic peptide; %WV, percentage of venom. (DOCX) [file pntd.0011564.s003.docx]

| **SPECIES** | **PLA_2_** | **SVSP** | **SVMP** | **LAAO** | **3FTx** | **KUN** | **CTL/SNACLEC** | **DIS** | **CRiSP** | **NP** | **%WV** | **Ref** |
| --- | --- | --- | --- | --- | --- | --- | --- | --- | --- | --- | --- | --- |
| *B. arietans* | 4.3 | 19.5 | 38.5 |  |  | 4.2 | 13.2 | 17.8 |  |  | 99.2 | [1] |
| *B. jararaca* | 3.7 - 20.2 | 13.7 - 28.6 | 10.3 - 35.6 | 7.2 - 8.0 |  |  | 9.4 - 9.6 | 0.2 - 7.0 | 2.4 - 2.6 | 16.4 - 22.6 | 95.6 - 100 | [2,3] |
| *C. rhodostoma* | 4.4 | 14.9 | 41.2 | 7 |  |  | 26.3 |  | 2.5 |  | 96.3 | [4] |
| *D. russelii* | 32.8 - 35 | 3.2 - 16 | 6.9 - 24.8 | 0.3 - 5.2 |  | 4.6 - 28.4 | 1.8 - 22.4 | 0 - 4.9 | 2 - 6.8 |  | 92.1 - 97.7 | [5,6] |
| *E. carinatus* | 10.9 - 25.7 | 0.3 - 5.6 | 27.1 - 45.4 | 0 - 8.7 |  |  | 14.5 - 33.0 | 0 - 14.0 | 0 - 5.6 |  |  | [7,8] |
| *E. ocellatus* | 8.5 | 1.7 | 72.4 | 1.4 |  |  | 6.5 |  | 0.3 |  | 93.5 | [9] |
|  |  |  |  |  |  |  |  |  |  |  |  |  |
| *B. multicinctus* | 56.1 - 66.4 |  | <0.1 - 0.8 | 0.16 - 2.1 | 27.5 - 32.6 | 0.5 - 2.3 |  |  | 2.4 | <0.1 | - | [10,11] |
| *N. haje* | 4.0 |  | 9 | 1.0 | 60.0 | 1.9 |  |  | 10.0 |  | 85.9 | [12] |
| *N. mossambica* | 27.1 |  | 2.6 |  | 69.3 |  |  |  |  |  | 99 | [13] |
| *N. naja* | 11.4 - 21.4 | 0 - 0.3 | 0.9 - 1 | 0 - 0.8 | 63.8 - 80.5 | 0 - 0.4 |  |  | 2.1 - 3.7 | 0 - 2.0 | 79.8 - 99.1 | [14,15] |

References

1. Calvete JJ, Escolano J, Sanz L. Snake venomics of Bitis species reveals large intragenus venom toxin composition variation: Application to taxonomy of congeneric taxa. J Proteome Res. 2007;6: 2732–2745. doi:10.1021/pr0701714

2. Gonçalves-Machado L, Pla D, Sanz L, Jorge RJB, Leitão-De-Araújo M, Alves MLM, et al. Combined venomics, venom gland transcriptomics, bioactivities, and antivenomics of two Bothrops jararaca populations from geographic isolated regions within the Brazilian Atlantic rainforest. J Proteomics. 2016;135: 73–89. doi:10.1016/J.JPROT.2015.04.029

3. Sousa LF, Nicolau CA, Peixoto PS, Bernardoni JL, Oliveira SS. Comparison of Phylogeny, Venom Composition and Neutralization by Antivenom in Diverse Species of Bothrops Complex. PLoS Negl Trop Dis. 2013;7: 2442. doi:10.1371/journal.pntd.0002442

4. Tang ELH, Tan CH, Fung SY, Tan NH. Venomics of Calloselasma rhodostoma, the Malayan pit viper: A complex toxin arsenal unraveled. J Proteomics. 2016;148: 44–56. doi:10.1016/j.jprot.2016.07.006

5. Kalita B, Patra A, Mukherjee AK. Unraveling the Proteome Composition and Immuno-profiling of Western India Russell’s Viper Venom for In-Depth Understanding of Its Pharmacological Properties, Clinical Manifestations, and Effective Antivenom Treatment. J Proteome Res. 2017;16: 583–598. doi:10.1021/acs.jproteome.6b00693

6. Tan NH, Fung SY, Tan KY, Yap MKK, Gnanathasan CA, Tan CH. Functional venomics of the Sri Lankan Russell’s viper (Daboia russelii) and its toxinological correlations. J Proteomics. 2015;128: 403–423. doi:10.1016/j.jprot.2015.08.017

7. Bhatia S, Vasudevan K. Comparative proteomics of geographically distinct saw-scaled viper (Echis carinatus) venoms from India. Toxicon. 2020;7: 100048. doi:10.1016/j.toxcx.2020.100048

8. Patra A, Kalita B, Chanda A, Mukherjee AK. Proteomics and antivenomics of Echis carinatus carinatus venom: Correlation with pharmacological properties and pathophysiology of envenomation. Sci Rep. 2017;7: 1–17. doi:10.1038/s41598-017-17227-y

9. Casewell NR, Harrison RA, Wüster W, Wagstaff SC. Comparative venom gland transcriptome surveys of the saw-scaled vipers (Viperidae: Echis) reveal substantial intra-family gene diversity and novel venom transcripts. BMC Genomics. 2009;10: 1–12. doi:10.1186/1471-2164-10-564

10. Ziganshin RH, Kovalchuk SI, Arapidi GP, Starkov VG, Hoang AN, Thi Nguyen TT, et al. Quantitative proteomic analysis of Vietnamese krait venoms: Neurotoxins are the major components in Bungarus multicinctus and phospholipases A2 in Bungarus fasciatus. Toxicon. 2015;107: 197–209. doi:10.1016/j.toxicon.2015.08.026

11. Shan LL, Gao JF, Zhang YX, Shen SS, He Y, Wang J, et al. Proteomic characterization and comparison of venoms from two elapid snakes (Bungarus multicinctus and Naja atra) from China. J Proteomics. 2016;138: 83–94. doi:10.1016/j.jprot.2016.02.028

12. Malih I, Ahmad rusmili MR, Tee TY, Saile R, Ghalim N, Othman I. Proteomic analysis of moroccan cobra naja haje legionis venom using tandem mass spectrometry. J Proteomics. 2014;96: 240–252. doi:10.1016/j.jprot.2013.11.012

13. Petras D, Sanz L, Segura Á, Herrera M, Villalta M, Solano D, et al. Snake venomics of African spitting cobras: Toxin composition and assessment of congeneric cross-reactivity of the Pan-African EchiTAb-Plus-ICP antivenom by antivenomics and neutralization approaches. J Proteome Res. 2011;10: 1266–1280. doi:10.1021/pr101040f

14. Dutta S, Chanda A, Kalita B, Islam T, Patra A, Mukherjee AK. Proteomic analysis to unravel the complex venom proteome of eastern India Naja naja: Correlation of venom composition with its biochemical and pharmacological properties. J Proteomics. 2017;156: 29–39. doi:10.1016/J.JPROT.2016.12.018

15. Sintiprungrat K, Watcharatanyatip K, Senevirathne WDST, Chaisuriya P, Chokchaichamnankit D, Srisomsap C, et al. A comparative study of venomics of Naja naja from India and Sri Lanka, clinical manifestations and antivenomics of an Indian polyspecific antivenom. J Proteomics. 2016;132: 131–143. doi:10.1016/J.JPROT.2015.10.007
