# Supplementary figures and images for: Development of a high-throughput in vitro screening method for the assessment of cell-damaging activities of snake venoms
Source: PLoS Negl Trop Dis. 2023 Aug 17;17(8):e0011564. doi: 10.1371/journal.pntd.0011564 (PMC10465002; doi:10.1371/journal.pntd.0011564)

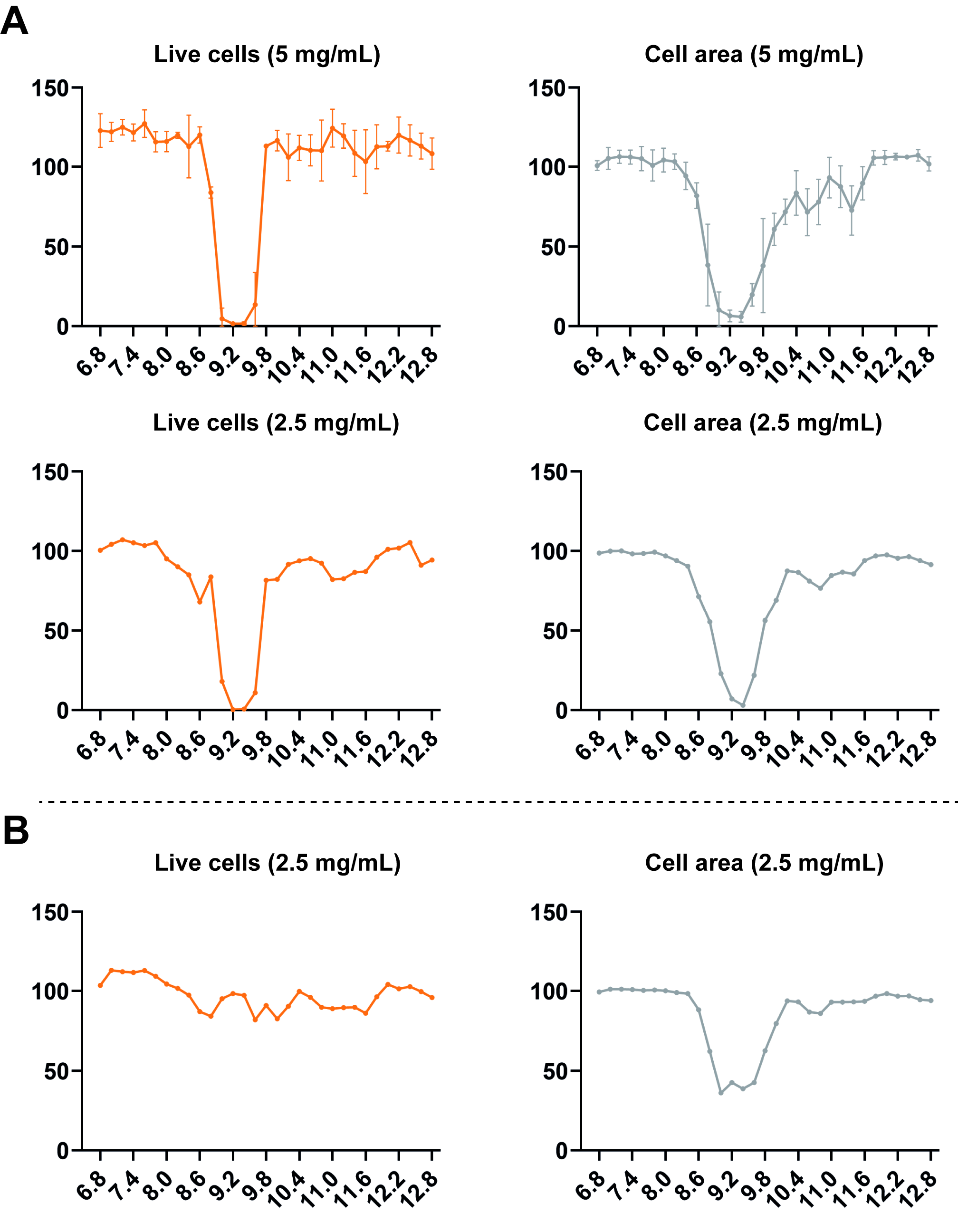

Supplement: S12 Fig — (A) Bioactivity profiles for venom concentrations 5.0 mg/mL and 2.5 mg/mL that were presented to the cells in an 80/20% ratio (i.e. 80% venom fractions combined with 20% growth medium. (B) Bioactivity profiles of venom (2.5 mg/mL) that was given to the cells in an 50/50% ratio. Bioactivity chromatograms obtained by plotting the results of two assays: live cell count (orange) and cell surface area (grey) The peaks with negative minima indicate the presence of bioactive compounds respectively. Time (in min) is represented on the X-axes and percentage relative to negative control is given on the Y-axes. Measurements are presented as the mean of three individual experiments (N = 3), error bars depict SD. Data of the 5 mg/mL fractions are presented as the mean of three individual experiments (N = 3), error bars depict SD; measurements of the bioactivity profiles for venom concentrations of 2.5 mg/mL are N = 1. (TIF) [file pntd.0011564.s016.tif]
